# Quality control of imbalanced mass spectra from isotopic labeling experiments

**DOI:** 10.1186/s12859-019-3170-1

**Published:** 2019-11-06

**Authors:** Tianjun Li, Long Chen, Min Gan

**Affiliations:** 1 Department of Computer and Information Science, University of Macau, Taipa, Macau, China; 2 College of Mathematics and Computer Science, Fuzhou University, Fuzhou, Fujian, China

**Keywords:** Mass Spectra, Proteomics, Imbalanced Data, Quality Control, Gradient Boosting

## Abstract

**Background:**

Mass spectra are usually acquired from the Liquid Chromatography-Mass Spectrometry (LC-MS) analysis for isotope labeled proteomics experiments. In such experiments, the mass profiles of labeled (heavy) and unlabeled (light) peptide pairs are represented by isotope clusters (2D or 3D) that provide valuable information about the studied biological samples in different conditions. The core task of quality control in quantitative LC-MS experiment is to filter out low-quality peptides with questionable profiles. The commonly used methods for this problem are the classification approaches. However, the data imbalance problems in previous control methods are often ignored or mishandled. In this study, we introduced a quality control framework based on the extreme gradient boosting machine (XGBoost), and carefully addressed the imbalanced data problem in this framework.

**Results:**

In the XGBoost based framework, we suggest the application of the Synthetic minority over-sampling technique (SMOTE) to re-balance data and use the balanced data to train the boosted trees as the classifier. Then the classifier is applied to other data for the peptide quality assessment. Experimental results show that our proposed framework increases the reliability of peptide heavy-light ratio estimation significantly.

**Conclusions:**

Our results indicate that this framework is a powerful method for the peptide quality assessment. For the feature extraction part, the extracted ion chromatogram (XIC) based features contribute to the peptide quality assessment. To solve the imbalanced data problem, SMOTE brings a much better classification performance. Finally, the XGBoost is capable for the peptide quality control. Overall, our proposed framework provides reliable results for the further proteomics studies.

## Background

Computational methods in proteomics are mainly designed to improve the analysis performance of MS. There are many well designed methods, like the molecular formulas predicting [1], the linear regression for overlapped ^18^O/^16^O ratio estimation [2], the statistical methods for corresponding feature identification [3], the self-boosted percolator for peptide prophet enhancing [4, 5] and the peptide identification for mixture spectra [6] to name a few.

In the Stable Isotope Labeling with Amino Acids in Cell Culture (SILAC) based proteomics experiments, the amino acids with *light* and *heavy* labels are metabolized into peptides [7]. Then the identified peptides show only a fixed shift in mass in different conditions of the spectra. However, the pairs of heavy-light peptide and some other features in SILAC data are often influenced by some biological, experimental or chemical errors. Since these errors may affect the later quantitative analysis, and the errors are hard to handle manually, there is a great need to have some computational methods to assess the spectral quality [8].

Some software or platforms have been provided to handle the whole peptide analysis workflow, such as OpenMS [9], MaxQuant [10] and Trans-Proteomic Pipeline (TPP) [11–13]. These software can be used to convert the raw data into the readable files with analysis results. The whole peptide analysis workflow is defined as follows: “raw data converting → sequence database identification → validation → quantification" [14]. Currently, researchers pay more attention to the validation or quality control of quantification part in the workflow, and many methods have been proposed to this end. For example, the signal-to-noise ratio is proven to be an essential factor in ratio estimations for the isotope labeling based experiments [15, 16]. In addition, the preceding peak is demonstrated to be useful when compared with the target peak in the same scan of the peptide [17]. These signal-to-noise ratios and preceding peak ratios are some mass profiles, and many studies have demonstrated the importance of controlling the quality of mass profiles [16] in quantification analysis.

**Fig. 1:**
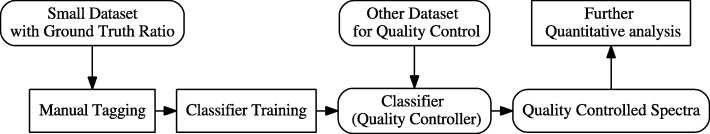
The design of quality controller. The spectra after peptide quality control can be used for further studies, such as quantification

Many methods have been conducted to control the spectral quality for better quantification results. These methods can be mainly divided into naive methods [18], classification methods [19, 20] and statistical methods [21], while the classification approach is the most widely used ones [19]. For details, some features are extracted from few LC-MS raw data, and then the corresponding quality tags are generated manually. The features and associated tags are formed together into a data set. We then divide the data set into a training set and a validation set, where the training set is used for classifier training and the validation set is used to ensure the classifier’s performance. Finally, we can evaluate the quality of other spectra by extracting the same features from related spectra and then passing through the trained classifier. The mass profiles classified as the high quality ones are retained for further analysis. The general diagram for the design of quality control is illustrated in Fig. 1.

There are pairs of heavy and light peptide peak clusters in LC-MS[2]. Most spectral feature extracting methods focus on related clusters in one scan. However, based on the extracted ion chromatogram (XIC), the information in the nearby scans also helps to quantitation [22]. This motivates us to derive four new features from the corresponding neighbor scans to construct the classifier. Combining nine features extracted from single scan, we totally release thirteen features as the inputs of classifiers.

To comprehensively consider all the features related to the quality of quantification, machine learning methods can be used for quality assessment of spectra, including the support vector machine (SVM) based models [19, 20] and the tree-structured models [23–25]. Note that proteomics also has some models based on deep neural network (DNN) [26–28], but DNN is time-consuming and requires a lot of training data to deal with the over-fitting problem, which is not suitable for quantification. So in this paper, we do not discuss deep models.

When training a classification model, by default most machine learning algorithms treat the training set as the balanced one. However, because learning system has difficulty in deriving concepts from the minority class, imbalanced data has become one of the major challenges affecting the performance of machine learning algorithms [29, 30]. The re-sampling method is one of the most important methods for dealing with data imbalance problem. In the field of re-sampling, there are two well-known approaches: the under-sampling one and the over-sampling one. But the under-sampling method may discard potentially relevant information, while the over-sampling method may increase the likelihood of over-fitting and the complexity of the model training [31]. Therefore, we need decent ways to deal with unbalanced data problems. SMOTE is one of the mature methods [29]. This method re-samples new data point by combining random factors from zero to one with its *k* nearest neighbors.

Considering the fact that there are only a few problematic spectra in LC-MS analysis, we carefully addressed the important problem of classifying imbalanced spectral dataset in this paper. We suggest using the SMOTE to handle the unbalanced data, and employing the famous XGBoost[32] as the classifier, which achieves outstanding performance without having high overhead in computation time. XGBoost is a kind of tree-structured model. The basic idea of the tree-structured model is to design an ensemble approach for several rule-based binary trees [33, 34]. In the past decades the *Gradient Boosting* [35] is the most famous tree ensemble method, and this technique led to the renowned Gradient Boosting Decision Tree (GBDT). XGBoost is a variant of GBDT, and it has gained the popularity by winning many machine learning competitions since its availability.

We evaluate the classifiers by the data with different heavy-light ratios. The SMOTE technique shows its capability in improving the performance of classifiers by re-balancing the data, and the SMOTE XGBoost shows its reliability in assessing the quality of mass profiles in LC-MS data.

## Results and discussion

In SILAC technique, two populations of cells are cultivated in cell culture at first. Then the growth medium with normal amino acids is fed to one population. On the contrary, the growth medium containing labeled heavy isotopes amino acids is fed to another one. The labeled amino acids are usually the lysine (K, +8.014199) and arginine (R, +10.008269). This population of cells would replace the heavy-labeled-isotopes into their proteins, so that the combined normal (light)-heavy cell populations can be analyzed together by LC-MS. The produced mass spectra can reflect the abundance ratios for the peptides and proteins in concern. In this study, the raw data is the combination of SILAC labeled yeast (S. cerevisiae) and unlabeled ones that mixed at various light/heavy ratios (1:2, 1:1, 1.5:1 and 2:1), and we analyzed these data by TPP in a web-based distributed system. The PeptideProphet [5] and the ASAPratio [36] are the TPP built-in validation and quantitation methods, respectively. We also called the TPP derived peptide ratios as the ASAPRatios, which will be used to tag the training data and evaluate the quality controller.

### Feature extraction and training data preparation

Considering the strong correlations between the spectral features and the quality of the spectra, we extract thirteen features to design the quality classifiers. These features are mass deviation (MassDev), signal to noise ratio (S/N), preceding peak ratio (PPR), six isotope deviations (IsoDevs) and four scan isotope pattern deviations (SIDs). These features are discussed in “Methods” section. The final processed data is formed by these features with the size of *n* ×13 for one sample, where *n* denotes the number of spectra.

To train a classifier, the data and corresponding training labels are required. The training label is intended to indicate the relationship between the target and the class. But in this study, there are two types of labels, one for training labels and the other for isotope labeling. Therefore, to avoid confusion, we adopt *tags* to represent the training labels.

During the whole procedure of LC-MS based proteomics experiments, the errors or mistakes are unavoidable, so it is desirable for us to filter out the low quality spectra (or corresponding peptides). If we have some historical spectral data with low or high quality tags, then we can use them directly to train the classifiers for quality control. However, we usually only have some LC-MS data with fixed mix ratios, and the samples in the data do not have quality tags like good (positive) or bad (negative). To prepare the quality tags, we assume that we can have some data with prefixed quantitative ratios, and we followed the work of [16, 20] to manually labeled the data for the training of classifiers via peptide quantitative ratios and mass accuracy (deviation). It should be clarified that the classifiers trained by the manually tagged data only accept the 13 features designed in this paper and they will not use the quantitative ratio as the input feature.

**Fig. 2:**
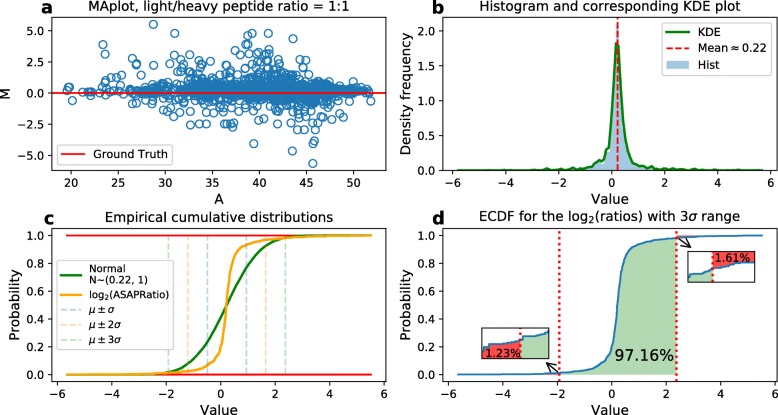
Data distribution in peptide 1:1 sample. **a**. MA plot of ASAPRatio. **b**. Histogram and corresponding KDE plot for the binary logarithmic ASAPRatio. **c**. Empirical CDF for the binary logarithmic ASAPRatio and one normal distribution with k- *σ* range given by Chebyshev’s Inequality, k=1,2,3. **d**. Cumulative probability within 3 *σ* range of the binary logarithmic ASAPRatio, the red dotted lines stand for the boundaries of [ *μ*±3*σ*]

In details, we use ASAPRatio to denote the estimated quantitative ratio, and the high quality peptides should have ASAPRatios be close to the ground truth. Figure 2 shows the details about the distribution of ASAPRatio in this 1:1 sample data. Figure 2a is the MA plot for the sample. The rest of the sub-figures (b, c and d) are generated by the binary logarithmic sample data (log2(Sample)), which are used for the probability density function (PDF) and cumulative distribution function (CDF). The MA plot for other samples are also included [see Additional file 1]. The histogram and the corresponding Gaussian kernel density estimation (KDE) in Fig. 2b represent the PDF of the sample. The empirical CDF and the Chebyshev’s Inequality[37] are shown in Fig. 2c. Known by Chebyshev’s Inequality, the data located in the range [ *μ*−3*σ*,*μ*+3*σ*] would keep at least 8/9 of the whole data. The empirical CDF illustrates that our sample has almost the same cumulative probability just in the locations that refer to 3*σ* when compared to the N ∼(0.22, 1) one. Figure 2d shows the CDF details with the interval [ *μ*−3*σ*,*μ*+3*σ*] in our data distribution. Hence, three-sigma of Chebyshev’s Inequality is applied to group the logarithm transformed ASAPRatios manually, and its result is marked as *ratio-tag*.

**Fig. 3:**
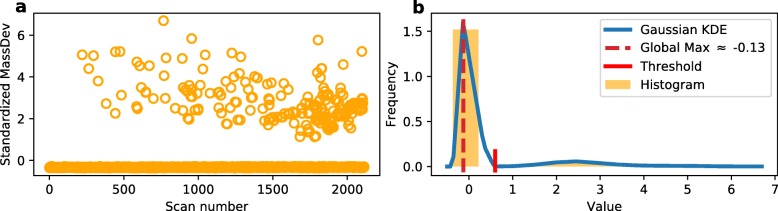
Distribution of mass deviation and corresponding histogram and density map. **a**. Distribution of mass deviation. **b**. Histogram and the density map of standardized mass deviation, the red vertical line refers the threshold

The *ratio-tag* based on ASAPRatio narrows down the scope of high quality data, but it is not accurate enough because the distorted low quality spectrum may also produce a correct ratio. However, there is an intuition that the identified high quality peak should adopt the mass value close to the theoretical one. Thus, the mass deviation can be used to group the data further by this intuition. Figure 3 shows the distribution of standardized ((*x*−*μ*)/*σ*) mass deviation and the corresponding histogram and density map as well. It follows from the histogram and density map in Fig. 3b that the standardized mass deviation value has a global maximum of about −0.13, which is the systematic bias of mass measurement. So a spectrum is tagged as positive if the standardized mass deviation value fell in the interval [−0.13−threshold,−0.13+threshold]. According to the distribution of mass deviations, here the threshold is set to 0.5 to exclude all the outliers. The corresponding threshold is also plotted as a short red vertical line in the density map. This tag result based on mass deviation is marked as *mass-tag*.

For the final tags of the 1:1 sample, the spectra with both positive *ratio-tag* and positive *mass-tag* are marked as positive, while the others are regarded as negative ones. Now we have a training data set for the optimization of the classifier. Since the quantitative ratios are not used as input feature, the trained classifiers will not be in favor of them. For the feature of mass deviation, the bias to it may be a problem. But according to the experimental results, such a biasing does not happen. The features other than mass deviation used in this paper play important roles in the classifiers (See Table 2 for details), and this makes the trained models much better than simple mass deviation based quality controller (Please refer Fig. 3a).

### Over-sampling for the imbalanced data

Since there would be some inevitable contaminants in the process of culturing amino acids or errors occurred in the data collection, the final data would have some errors or unreliable parts. More importantly, these contaminants or errors are rare and unpredictable. But for the whole data, there is only few spectra influenced by these unreliable parts, theoretically. So the quantity of the high quality peptides (Positive) has an imbalance ratio with the low quality ones (Negative). By checking the tags of the training set (75% of total training data), we find that the number of positive tags is six times larger than that of the negative ones. The domination of positive tags highlights the data imbalance problem, which may lead to result the classifier does not learn enough from the minority class when training.

We apply the SMOTE to re-balance the training set. Table 1 shows the details of the quantity changes in this training sample.

**Table 1:** Quantity changes with or without resample methods for the training set

	Positive Number	Negative Number	Total Number (for training)
Original Data	1585	529	2114
After Split (75%)	1367	218	1585
SMOTE	1367	**1367**	**2734**

The bold ones are the ones changed by the resample methods

**Table 2:** Gain as the feature importance for the XGBoost model with or without SMOTE

Feature Name	Gains without SMOTE	Gains with SMOTE
MassDev	11.54877	141.50188
PPR	0.52670	3.73862
S/N	0.39289	7.49105
IsoDev_Light1	1.30760	30.78152
IsoDev_Light2	2.51584	6.70143
IsoDev_Light3	3.52582	2.30519
IsoDev_Heavy1	0.64070	4.80937
IsoDev_Heavy2	0.49069	4.60379
IsoDev_Heavy3	0.69931	7.06721
SID_sum_	0.25840	3.94553
SID_0_	0.60187	5.18034
SID_1_	0.62181	5.15330
SID_2_	0.54873	4.74491

We also adopt the “gain” as the indicator of feature importance for the trained XGBoost models in Table 2. The gain is the most relevant attribute to interpret the relative importance of each feature in XGBoost. It implies the relative contribution of the corresponding feature to the model and is calculated by taking each feature’s contribution for each tree in the model. Higher gain means this feature contributes more for prediction, and the gain with very small value usually means that the contribution of this feature is not significant.

Generally, a classifier can be considered appropriate if all meaningful training features contribute to the classifier. In this study, all the features in the training set were extracted based on the nature of the peptide, which is valuable for the peptide quantification. However, it follows from the Table 2 that the model generated from original data has many very small feature gains, so we think that the model may not be learned sufficiently because there are some features that contribute only little to it.

### Classifier training and validation

There are many parameter tuning methods, and randomized search or grid search may be the most basic automatic methods for the models without deep architecture. However, these methods are really time-consuming and limited by the predefined set of parameter grids. In this paper, we employ a Bayesian optimization tool[38] to tune the XGBoost model, and the XGBoost model is implemented by the xgboost python package[39]. In details, we randomly divide the total training data into one training set (75%) and one testing set (25%), and then apply the training set to the optimization tool with XGBoost model for training. The optimization aims to find out the parameters that have the maximum mean value of the 10-fold cross validation evaluation scores under different parameters. We set the “roc_auc" value as the evaluation score of the cross validation. The bounds of the parameters in the optimization tool are set as follows:

- “learning_rate": (0.01, 0.3),
- “n_estimators": (10, 2000),
- “max_depth": (3, 10),
- “gamma": (0, 0.05),
- “colsample_bytree": (0.7, 1),
- “subsample": (0.7, 1).

The first three parameters denote the structure of the model, and the last three parameters solve the over-fitting problem by controlling the complexity and robustness of the model. Note that the values in “n_estimators" and “max_depth" are set as integer values.

After 30 iterations of the Bayesian optimization, we have got the following parameters:

- “learning_rate": 0.197,
- “n_estimators": 11,
- “max_depth": 10,
- “gamma": 0.04,
- “colsample_bytree": 0.97,
- “subsample": 0.96.

The trained classifiers can be applied to other samples without manual tagging. If the ground truth peptide ratio is provided, then the favorable classifiers should find high quality spectra that possess estimated heavy-light peptide ratios compactly close to the ground truth ones. Therefore, estimated peptide ratios are used to validate and evaluate classifiers.

**Fig. 4:**
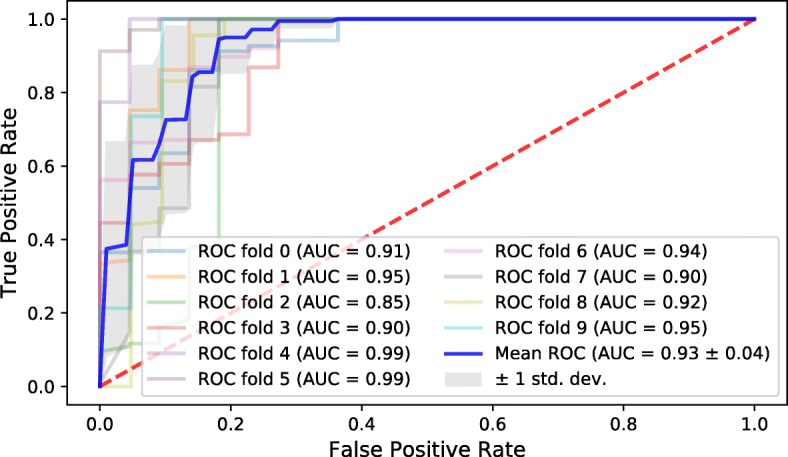
Cross validation with ROC curves and AUC values

To validate the performance of the classifier, the receiver operating characteristics (ROC) curve is employed. Specifically, since we applied the over-sampling in training, the 10-fold cross validation in parameter tuning is slightly different. The traditional cross validation method divides the training data evenly into *k* folds, and then enumerates *k* times as follows: each time we select one fold as the testing fold, and the other *k*−1 folds are used as the training folds. Then the classifier is trained by the training folds, and we evaluate the classifier using the testing fold. For this special cross validation, we added the over-sampling for the *k*−1 training folds in the enumeration, and then the classifier is trained by the over-sampled training folds. Note that the testing fold is not over-sampled. By this manner, we validated the classifier by 10-fold cross validation with ROC curve and area under ROC curve (AUC) values in Fig. 4.

### Quality control results

The XGBoost classifier is trained by the SMOTE re-balanced data with the parameters tuned above. For comparison approach, we added the SVM based quality control framework[20] as the baseline method. The features and the parameters in this SVM based framework are the same as the ones mentioned in [20]. Specially, a class weight parameter is declared in SVM for the imbalanced problem, and the imbalance ratio in their case is 2.2. For our situation, the imbalance ratio is about 6. Hence, we changed the default weight parameter 2.2 to 6. The SVM baseline method is implemented by the svm package in python scikit-learn[40].

We evaluated our classifiers on four SILAC yeast samples (1:2, 1:1, 1.5:1 and 2:1) [see Additional file 2]. Note that only a part of the 1:1 sample was used for training, and here the entire 1:1 one was used for evaluation. Furthermore, we adopted the mean, the mode and the coefficient of variation (CV) as the evaluation criteria. The criteria are calculated by the binary logarithmic peptide ratios. Typically, CV is defined as *CV*=*σ*/*μ*. But for the logarithmic data, the way to calculate CV should be changed to make sense [41, 42], that is

1 $$\begin{array}{@{}rcl@{}} CV &=& {\sqrt{\exp{([\ln(\text{base})]^{2}\sigma^{2})}-1}} \\ &=& {\sqrt{\text{base}^{[\ln{(\text{base})}]\sigma^{2}}-1}}. \end{array} $$

While in our study, since the base of the logarithm is 2, we use this to calculate CV: $CV = \sqrt {{2}^{(\ln {2})\sigma ^{2}}-1}$.

**Fig. 5:**
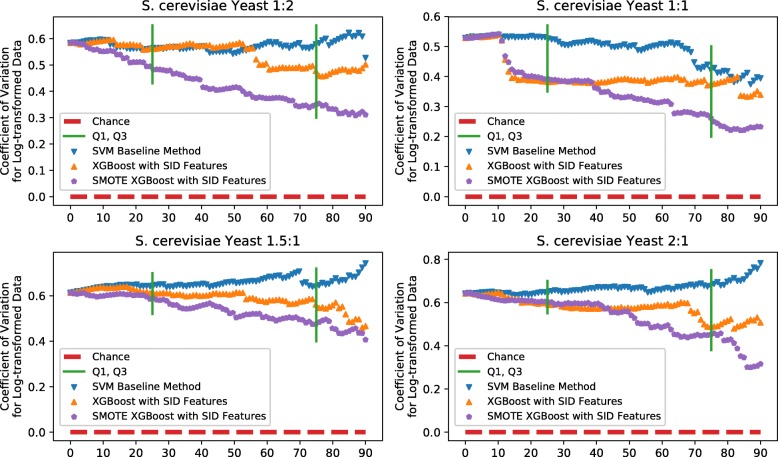
Coefficient of ratio variation as a function of the percentage of spectra filtered by SVM baseline method (blue triangle), XGBoost without SMOTE method (orange triangle) and proposed XGBoost method (purple pentagon) for four yeast samples: 1(L):2(H), 1(L):1(H), 1.5(L):1(H) and 2(L):1(H). The green vertical lines denote for Q1 and Q3. The improvement of the CV’s obtained by using XGBoost becomes fairly reliable

Figure 5 illustrates the overall performance of our XGBoost models and SVM baseline model using CV as the indicator. It is clear that the quality of the peptide controlled by XGBoost model is quite concentrated compared to the SVM baseline approach, and this concentration is very useful for the quantitative analysis. Moreover, the classifier trained by the re-balanced data set provides better performance. It also can be seen from Figure 5 that it is more concentrated when the spectra are filtered out by about 30%. So we display the statistical details for the spectra that are filtered out by 30% in Table 3.

**Table 3:** Number of spectra, means, modes, and coefficients of variation for peptide ASAPRatios derived from four yeast samples (before filtering, after filtering by SVM base method, and after filtering by XGBoost model)

	Before filtering/After filtering by SVM baseline method/After filtering by XGBoost model			
Peptide ratio (lo*g*_2_(ratio))	Number of spectra	Mean	Mode	Coefficient of Variation
1:2 (-1)	2062/1444/1444	-0.77/-0.77/-0.80	-0.86/-0.89/-0.84	0.58/0.57/0.47
1:1 (0)	2114/1480/1480	0.22/0.22/0.21	0.19/0.19/0.19	0.53/0.51/0.39
1.5:1 (0.58)	2441/1709/1709	0.72/0.72/0.74	0.82/0.82/0.82	0.61/0.64/0.56
2:1 (1)	2110/1477/1477	1.09/1.07/1.12	1.30/1.29/1.30	0.64/0.65/0.59

It should be noted that the evaluation of control results just based on mean or mode is not reliable because the ASAPRatio is only the estimated peptide ratio and there is an unknown systematic bias in such kind of estimation. For example, one classifier A results in many ratios close to 1.1, and the other classifier B results in most ratios close to 1.2. Even we know the ground truth ratio is 1, we cannot conclude that the classifier A is better because the unknown systematic bias may be 0.2 which makes the estimated ratio more accurate when it is close to 1.2. So we mainly evaluate the control results based on the variance.

In another point of view, what we needed in quantitation is a more accurate quantitative result. For a set of quantified peptide ratios, we believe that the results are accurate if the ratios are well concentrated and distributed around a certain value. So the means and the modes in Table 3 refer the “certain value", while the CVs imply the concentration, and the CV value close to zero indicates more concentration. The means and the modes in the table are all close to the ground truth, and the CVs with XGBoost model are closer to zero than others. This means that the spectra quality controlled by XGBoost are more reliable. This method also provides reliable results for higher ratio samples [see Additional file 3].

#### Quality control for protein level quantification

Furthermore, the protein level quantification can benefit from the quality control of spectra. There is a basic idea that one protein should contain many peptides, the ratios in protein may vary with or without peptide quality assessment. The protein ratio should be compactly close to the ground truth when only using high quality spectra.

**Fig. 6:**
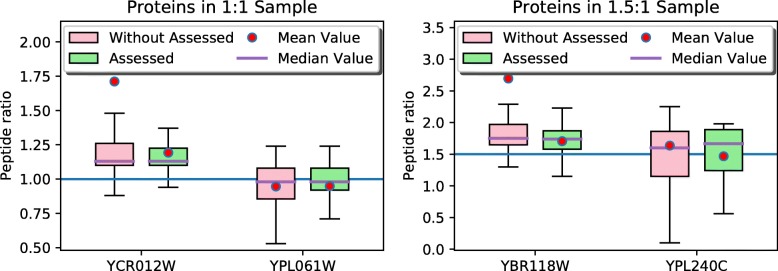
Box plot for ratio estimations of four proteins with or without quality assessor

We conduct a simple experiment on this basic idea and show the ratio changes of four proteins in two samples in Table 4. Figure 6 shows the box plot of ratios for the four proteins. The results show that the quality control method makes the estimated protein ratios close to the ground truth with smaller variances.

**Table 4:** Comparison of protein ratio estimations with or without peptide quality control

Protein	YCR012W 1:1 Sample		YPL061W 1:1 Sample		YBR118W 1.5:1 Sample		YPL240C 1.5:1 Sample	
Assessor	Pep. Num.	Pep. Ratio	Pep. Num.	Pep. Ratio	Pep. Num.	Pep. Ratio	Pep. Num.	Pep. Ratio
Without assessor	153	1.7109 ±3.6754	27	0.9459 ±0.3516	68	2.6947 ±7.4300	33	1.6364 ±1.0504
XGBoost assessor	131	1.1922 ±0.3274	25	0.9484 ±0.3636	28	1.7054 ±0.3479	4	1.4675 ±0.5568

## Conclusion

For better quantitative analysis in LC-MS based proteomics experiments, this paper introduces some new approaches to construct a reliable quality assessor of spectra for isotopic labeled samples. There are mainly four types of variation have been associated with ratio estimation [43], and this work mainly focuses on reducing the artificial variation.

We find that the peptide quantification may be influenced by the XIC, so we introduce new features based on nearby LC scans for better classification. We also notice that the unbalanced data may affect the results of the assessment. For this problem, we re-sample the unbalanced spectral features using SMOTE technique and train the classifiers using the SMOTE set. The trained classifiers are tested on SILAC labeled samples. The results show that SMOTE XGBoost classifier is the state-of-the-art and capable of the quality assessment for mass spectra.

The recently proposed new re-sample methods [44] can be considered in future work. Furthermore, the feature extraction functions and the pre-trained classifiers of this method can be easily embedded into the LC-MS based quantitative proteomics analysis pipeline.

## Methods

### Spectral features

#### Mass deviation

Theoretically, the mass of one peptide is a definite value by the components of its amino acid. We marked this definite value as the neutral peptide mass (M_*t*_). However, the experimental mass value would be different from the theoretical one due to the isotope. Meanwhile, we also marked the experimental peptide mass as M_*e*_, and typically this M_*e*_ is the precursor neutral mass (monoisotopic mass).

The mass deviation (MassDev) is defined as the deviation level of the mass value, which is shown by the following Eq. 2.

2 $$\begin{array}{@{}rcl@{}}  \text{MassDev} = \frac{\mathrm{M}_{t}-\mathrm{M}_{e}}{\mathrm{M}_{t}} \times 10^{6}, \end{array} $$

where 10^6^ is the unit parts per million (PPM), and smaller MassDev value refers better quality of the peptide.

#### Preceding peak ratio

The preceding peak of current peak has been explained to be an essential factor in evaluating the profile of a peptide in [17]. In details, the estimation of the ratio is unreliable when the preceding peak (M_*pp*_) is comparable to the mono-isotopic peak (M_*mp*_) in peak intensity. Hence, the reliability can be represented by the rate of M_*pp*_ and M_*mp*_, and this is the preceding peak ratio (PPR). PPR is defined in Eq. 3.

3 $$\begin{array}{@{}rcl@{}} \text{PPR} = \frac{\mathrm{M}_{pp}}{\mathrm{M}_{mp}}.  \end{array} $$

Note that the values would be set to zero if there are rare preceding peaks that cannot be identified, and the close to one PPR is a signal of unreliable spectra.

#### Signal to noise ratio

In signal processing, what we expected is the pure true peak signal. But noise is unavoidable now. In this way, the signal with less noise should be better, and one simple way to denote this is signal-to-noise ratio (S/N). The S/N is known as an important factor for evaluating the estimation ratio accuracy [15, 16], because the peptide with better quality usually has lower noise. Generally, the median value of peak intensities is set to the noise level, and the mono-isotopic peak intensity value indicates the signal level [45].

#### Isotope deviations

Isotope is the key to the labeling technique. The theoretical isotopic pattern is a set of values associated with the relative abundance of the isotopes, but the experimental one may deviate from it. So the isotope deviation (IsoDev) is another critical feature for the SILAC spectra and can be obtained by both light and heavy labeled peptides.

Suppose that TP represents the theoretical isotopic pattern and EP stands for the experimental one, then the definition of the isotope deviation is given by the following Eq. 4 [20],

4 $$\begin{array}{@{}rcl@{}} \text{IsoDev}_{i} = \frac{\text{TP}_{i}}{\text{TP}_{0}} - \frac{\text{EP}_{i}}{\text{EP}_{0}},  \end{array} $$

where *i*=1,2,3 are the different deviations in each pattern. Note that the TP_0_ and EP_0_ represent the abundance of theoretical peak and mono-isotopic abundance in experiment, respectively.

#### Scan isotope pattern deviations

**Fig. 7:**
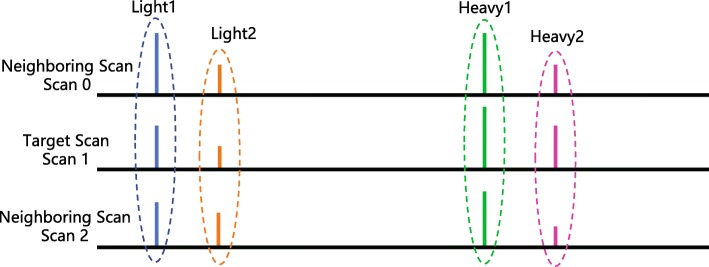
Example for the target scan and its corresponding neighboring scans. The blue lines indicate the first light peak intensities (Light1), the orange lines stand for the second light peak intensities (Light2), the green lines are the first heavy peak intensities (Heavy1) and the purple ones represent the second heavy peak intensities (Heavy2)

As far as we know, the accuracy of the estimated ratios is also influenced by the nature of corresponding peptides. While in a mass spectrometer the target peptide is identified at one corresponding LC scan, due to the continuity of LC and XIC, the neighboring scans of the target scan would also have valuable information, which is shown in Fig. 7.

The isotope patterns in neighboring scans are of great importance because they should be similar to the theoretical pattern of identified peptide. By considering the ratio between the first and the second peaks in the isotope clusters for the heavy and light peptides, we define a group of features named Scan Isotope Pattern Deviations (SIDs) to show the deviation between the mono first-second peak ratio of the target scan (M_0_) and the integration of the experimental first-second peak ratios of neighboring scans by (5),

5 $$\begin{array}{@{}rcl@{}} \text{SID}_{i} = \frac{\mathrm{E}_{i} - \mathrm{M}_{0}}{\mathrm{M}_{0}},  \end{array} $$

where

$$\begin{array}{@{}rcl@{}}  \mathrm{E}_{i} = \frac{\mathrm{L}2_{i}+\mathrm{H}2_{i}}{\mathrm{L}1_{i}+\mathrm{H}1_{i}}. \end{array} $$

Here *i*=0,1,2 refer the target and neighboring scans: scan0, scan1 and scan2. The L1_*i*_ and L2_*i*_ stand for the corresponding scan’s first and second light peak intensities (blue and orange peaks in Figure 7), respectively. Similarly, H1_*i*_ and H2_*i*_ are the heavy ones (green and purple peaks in Fig. 7), respectively.

In addition, the SI*D*_*sum*_ is designed to show the ratio between the summarized first and second peaks in the isotopic cluster from neighboring scans in (6),

6 $$\begin{array}{@{}rcl@{}} \mathrm{SID_{sum} = \frac{E_{sum}-M_{0}}{M_{0}}},  \end{array} $$

here

$$\begin{array}{@{}rcl@{}}  \mathrm{E_{sum} = \frac{\sum\limits_{i=0}^{2}(L2_{i}+H2_{i})}{\sum\limits_{i=0}^{2}(L1_{i}+H1_{i})}.} \end{array} $$

Due to the similarity among the isotope patterns in neighboring scans, the SIDs designed above should be close to each other and close to zero in high quality spectra. Similar to the PPR, there will be some unidentified peaks,in which case the SIDs would be set to -1. Therefore, the four SIDs are inserted into the feature set for training the classifiers.

### Synthetic minority over-sampling technique

**Fig. 8:**
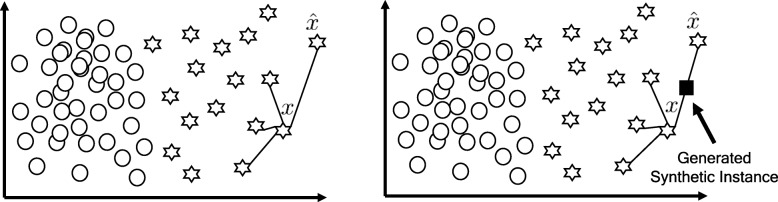
Example for SMOTE. The circles are the majority class samples and the hexagrams are the minority ones. The rectangle is the new sample generated by SMOTE

SMOTE is designed as a kind of over-sampling technique. Traditional over-sampling methods randomly repeat the minority samples as the newly-generated ones. But SMOTE calculates the nearest *k*^*th*^ neighbors by some distance methods at first, and then adds new sample between a data and it neighbors. More specifically, SMOTE adds a new data point by Eq. 7,

7 $$\begin{array}{@{}rcl@{}} x_{new} = x + rand(0,1) \times ||\hat{x}-x||,  \end{array} $$

where *x* denotes the minority class sample, ||.|| is the distance function and $\hat {x}$ represents the neighbors of *x*. Figure 8 illustrates the sampling procedure of SMOTE. We use the SMOTE in python environment by package *imbalanced-learn*[46].

### Extreme gradient boosting machine

In the field of supervised learning, gradient boosting[35] has been empirically verified to be effective. XGBoost is a kind of gradient boosting method with tree ensemble approach. This method has become very famous in Kaggle since 2014 and is known for its high performance and excellent results. In this algorithm, the following Eq. 8 gives the definition of *K* additive function ensemble model (*K* trees),

8 $$\begin{array}{@{}rcl@{}} \hat{y}_{i} = \sum\limits_{k=1}^{K}f_{k}(x_{i}), ~f_{k}\in\mathcal{F},  \end{array} $$

where *x*_*i*_ stands for the *i*^*th*^ sample, $\mathcal {F}$ is the space that containing all regression trees and *f*_*k*_ refers the *k*^*th*^ function in the functional space $\mathcal {F}$.

To train the ensemble model, the objective in (9) needs to be minimized,

9 $$\begin{array}{@{}rcl@{}} \mathcal{L}(\phi) = \sum\limits_{i=1}^{n}loss(y_{i},\hat{y}_{i}) + \sum\limits_{k=1}^{K}\Omega(f_{k}).  \end{array} $$

Here *loss* is a loss function that measures the difference between target *y*_*i*_ and prediction $\hat {y}_{i}$. The *Ω* penalizes the complexity and is defined in [32] in (10).

10 $$\begin{array}{@{}rcl@{}} \Omega(f) = {\gamma}T + \frac{1}{2}\lambda\sum\limits_{j=1}^{T}||w||^{2}.  \end{array} $$

The number of leaves in tree is defined as *T*,*γ* stands for minimum loss reduction, *λ* is the weight of regularization, ||*w*|| represents the corresponding leaves’ score (L2 norm), and the Eq. 11 can be used here to define the tree *f*(*x*),

11 $$\begin{array}{@{}rcl@{}} f_{t}(x) = w_{q(x)}, ~w{\in}R^{T},~q:R^{d}{\to}{1,2,\ldots,T},  \end{array} $$

here *w* denotes the score function, and *q* is the function that assigns each data point to the corresponding leaf (tree structure).

The objective in (9) is optimized by training the tree ensemble model in an additive (boosting) manner. Suppose that ${\hat {y}_{i}}^{(t)}$ is the prediction of the *i*^*th*^ instance at *t*^*th*^ training round, in additive manner a new *f*_*t*_ should be added to minimize the following objective,

12 $$\begin{array}{@{}rcl@{}} \mathcal{L}^{(t)} = \sum\limits_{i=1}^{n}loss\left(y_{i},\hat{y}_{i}^{(t-1)}+f_{t}(x_{i})\right) + \Omega(f_{t}).  \end{array} $$

Taylor expansion is applied to (12) to quickly optimize the objective in general setting [47], obtaining Eq. 13 here,

13 $$\begin{array}{*{20}l} \mathcal{L}^{(t)}&\simeq \sum\limits_{i=1}^{n}\left[loss\left(y_{i},\hat{y}_{i}^{(t-1)}\right)+g_{i}f_{t}(x_{i})+\frac{1}{2}h_{i}f_{t}^{2}(x_{i})\right]  \\&+\Omega(f_{t}),  \end{array} $$

where $g_{i} = \partial _{\hat {y}^{(t-1)}}loss\left (y_{i},\hat {y}^{(t-1)}\right)$ denotes the statistics of first order gradient on the loss function and $h_{i} = \partial _{\hat {y}^{(t-1)}}^{2}loss\left (y_{i},\hat {y}^{(t-1)}\right)$ is the second order ones.

The constant can be removed for simplifying the objective function at step *t*, and we get

14 $$\begin{array}{*{20}l} \mathcal{\tilde{L}}^{(t)} &= \sum\limits_{i=1}^{n}\left[g_{i}f_{t}(x_{i})+\frac{1}{2}h_{i}f_{t}^{2}(x_{i})\right] +{\gamma}T  \\ &+ \frac{1}{2}\lambda\sum\limits_{j=1}^{T}||w||^{2}.  \end{array} $$

Let *I*_*j*_={*i*|*q*(*x*_*i*_)=*j*} be the instance set of leaf *j*, then the Eq. 14 can be expanded to obtain

15 $$\begin{array}{*{20}l}  \mathcal{\tilde{L}}^{(t)} &=\sum\limits_{j=1}^{T}\left[\left(\sum\limits_{i{\in}I_{j}}^{}g_{i}\right)w_{j}+\frac{1}{2}\left(\sum\limits_{i{\in}I_{j}}^{}h_{i} + \lambda\right){w_{j}}^{2}\right]\\ &+ \gamma{T}.  \end{array} $$

Suppose that *q*(*x*) is a fixed structure, then the best weight $w_{j}^{*}$ of leaf *j* is calculated as follows,

16 $$\begin{array}{@{}rcl@{}} w_{j}^{*} = -\frac{G_{j}}{H_{j}+\lambda},  \end{array} $$

where $G_{j} = \sum \nolimits _{i\in {I_{j}}}g_{i}$ and $H_{j} = \sum \nolimits _{i\in {I_{j}}}h_{i}$, and the corresponding optimal value can be calculated by

17 $$\begin{array}{@{}rcl@{}} \mathcal{\tilde{L}}^{(t)}(q) = -\frac{1}{2}\sum\limits_{j=1}^{T}\frac{G_{j}^{2}}{H_{j}+\lambda} + \gamma{T}.  \end{array} $$

The quality of the tree structure can be evaluated by the score function (17), but it is impossible to enumerate all structures. So a greedy approach[35] is employed here. More specifically, we grow the tree from a single leaf and try to optimize (17) by splitting one leaf into two iteratively. For one split, the instance set is partitioned into left nodes(*IL*) and right nodes(*IR*) with *InstanceSet=IL∪IR*. So the gain $\mathcal {G}$ after this split is given by Eq. 18.

18 $$\begin{array}{*{20}l} \mathcal{G}_{split} &= \frac{1}{2}\left[\frac{G_{IL}^{2}}{H_{IL} + \lambda} + \frac{G_{IR}^{2}}{H_{IR} + \lambda} - \frac{(G_{IL}+G_{IR})^{2}}{H_{IL}+H_{IR} + \lambda}\right]  \\&-\gamma.  \end{array} $$

The splitting procedure will continue until the split gain $\mathcal {G}$ no longer positive.

## Supplementary information

**Additional file 1** This is a pdf file (233KB) containing all samples’ MA plots and corresponding distribution plots. The related peptide ratios are shown in the title of each plot.

**Additional file 2** This is a four-sheet xls file (7180KB) containing the TPP analysis results and extracted features, each sheet refers to one dataset with special ratio in its sheet name, e.g., 1_1 is the ratio 1:1 sample data.

**Additional file 3** This is a pdf file (69KB) containing an example for controlling the quality of the spectrum with high peptide ratio.

## Data Availability

The extracted data supporting the conclusions of this article is included within the article and its additional files. The raw data and codes used during the current study are available from the corresponding author on reasonable request.

## Abbreviations

AUC: Area under ROC curve
DNN: Deep neural network
GBDT: Gradient boosting decision tree
IsoDev: Isotope deviation
LC-MS: Liquid chromatography-mass spectrometry
MassDev: Mass deviation
PPM: Parts per million
PPR: Preceding peak ratio
ROC: Receiver operating characteristics
S/N: Signal to noise ratio
SID: Scan isotope pattern deviation
SILAC: Stable Isotope Labeling with Amino Acids in Cell Culture
SMOTE: Synthetic minority over-sampling technique
SVM: Support vector machine
TPP: Trans-Proteomic Pipeline
XGBoost: Extreme gradient boosting machine
XIC: Extracted ion chromatogram
