# Supplementary material for: Quality control of imbalanced mass spectra from isotopic labeling experiments
Source: BMC Bioinformatics. 2019 Nov 6;20:549. doi: 10.1186/s12859-019-3170-1 (PMC6833298; doi:10.1186/s12859-019-3170-1)
Supplement: Supplementary file 1 — Additional file 1 This is a pdf file (233KB) containing all samples’ MA plots and corresponding distribution plots. The related peptide ratios are shown in the title of each plot. [file 12859_2019_3170_MOESM1_ESM.pdf]

MA plot for the rest of the samples

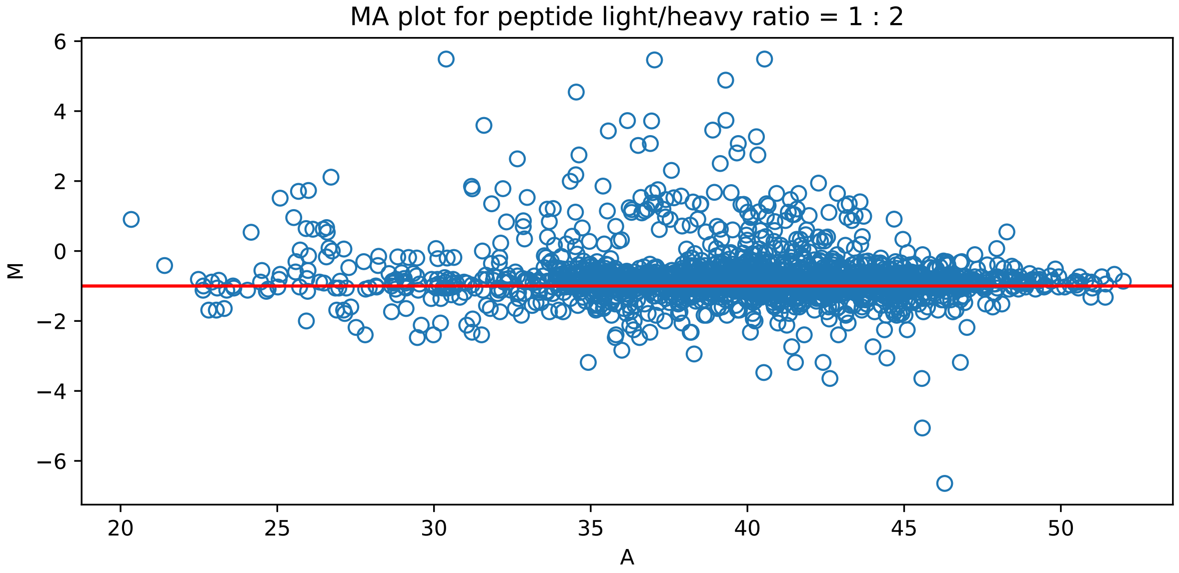

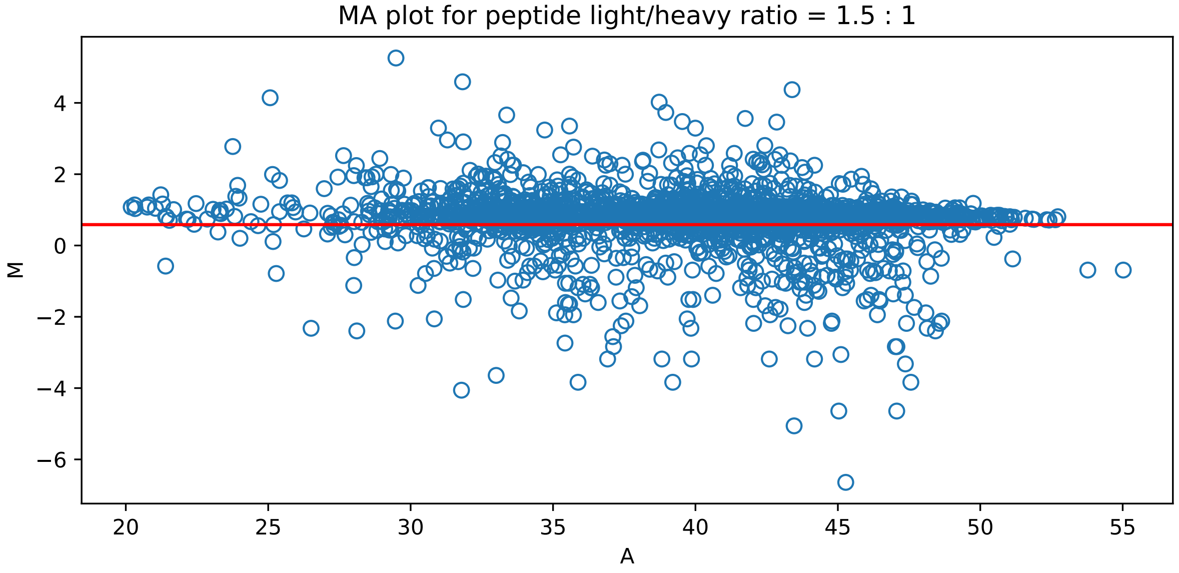

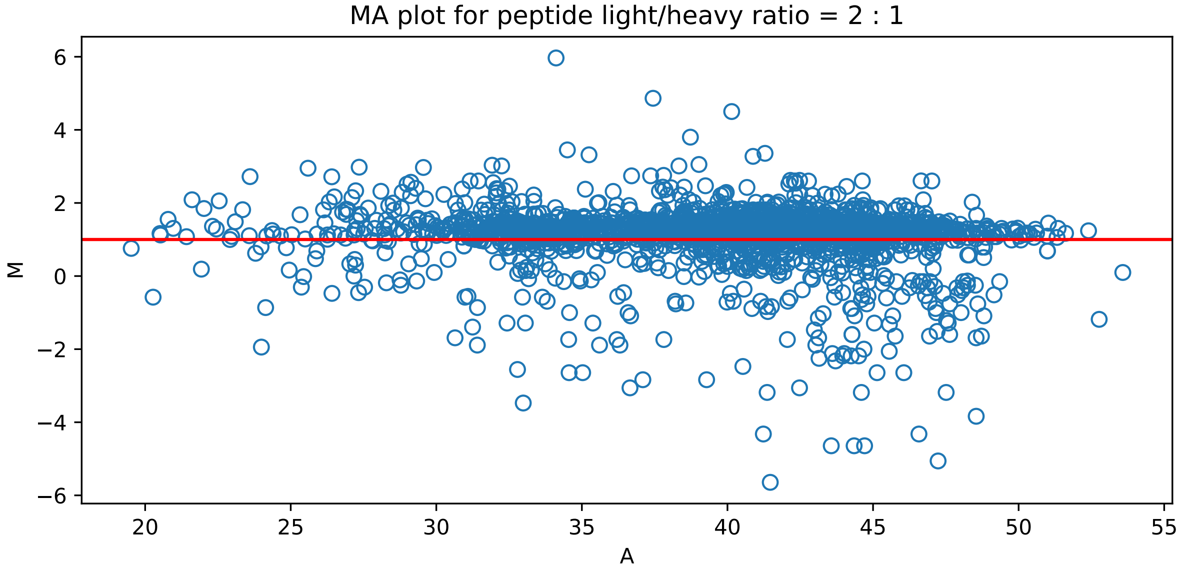

Distributions for $\log_2$(ASAPRatio)

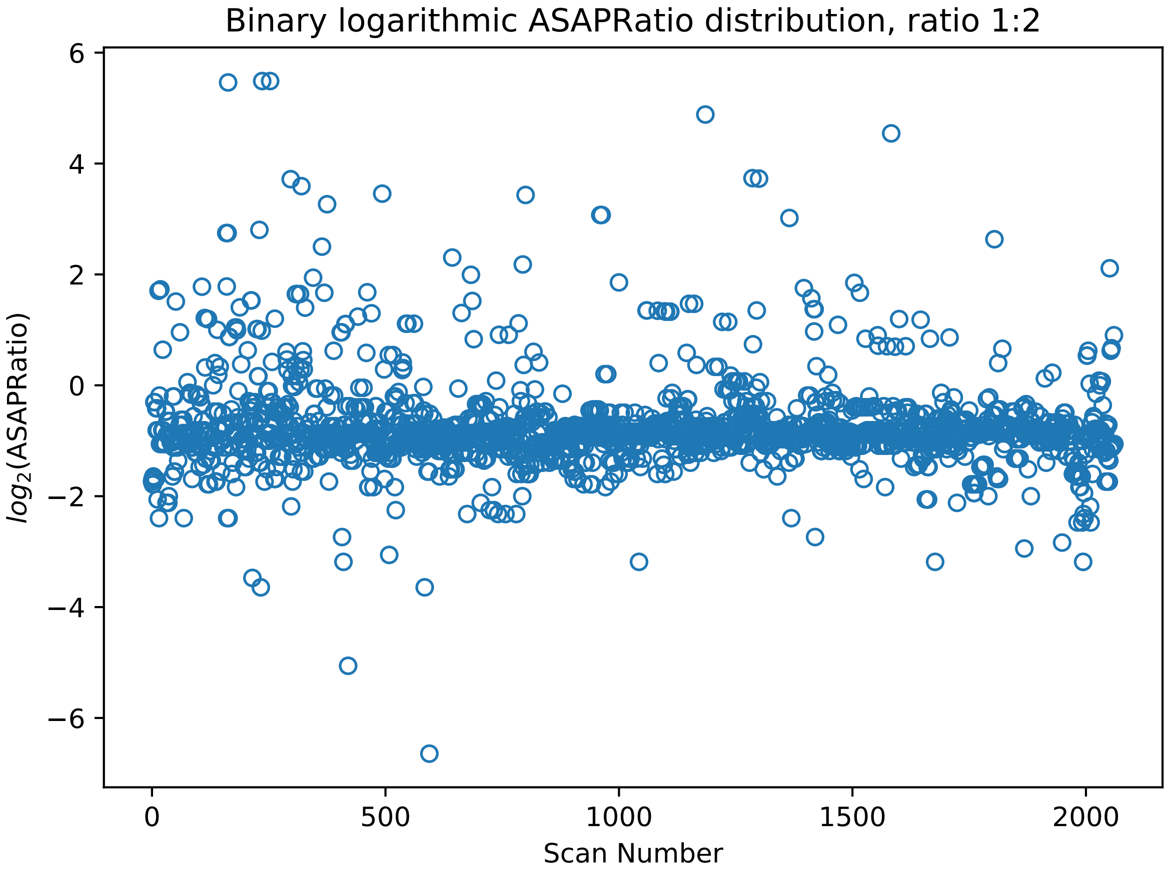

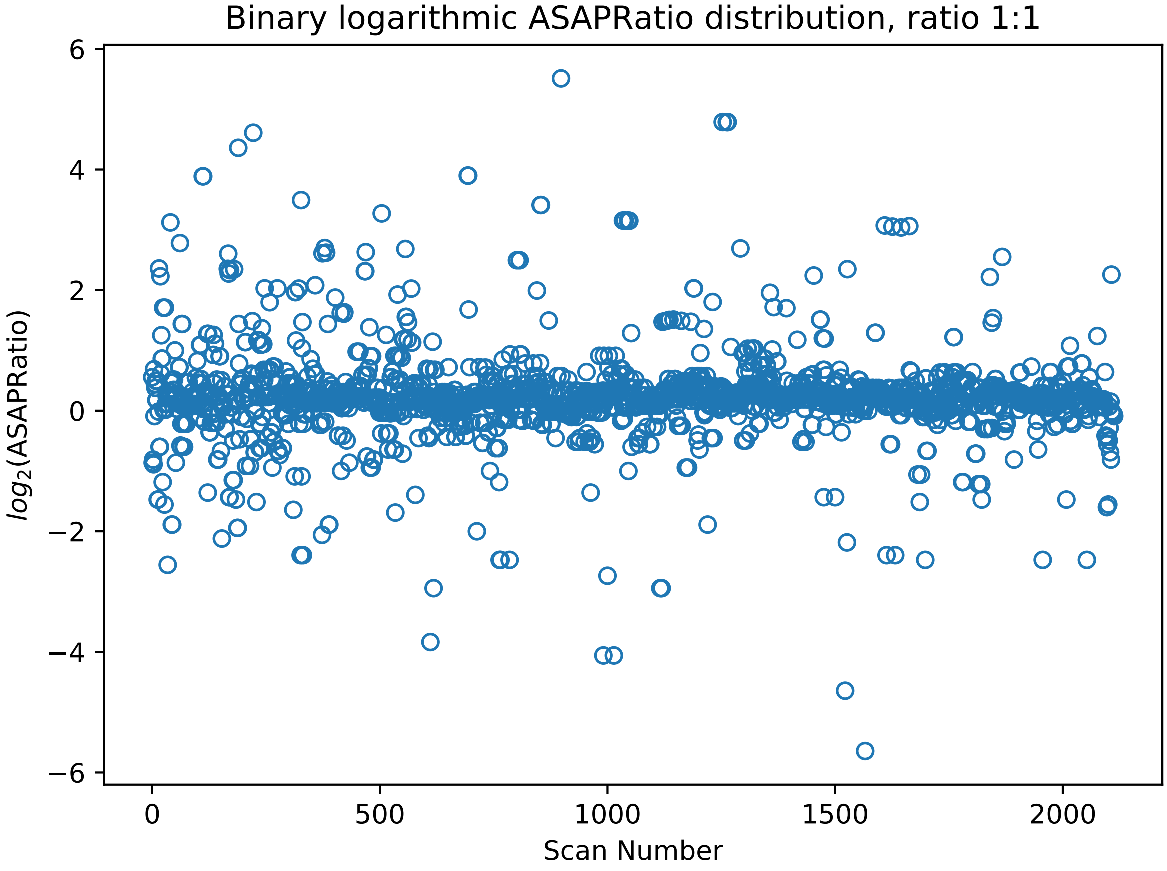

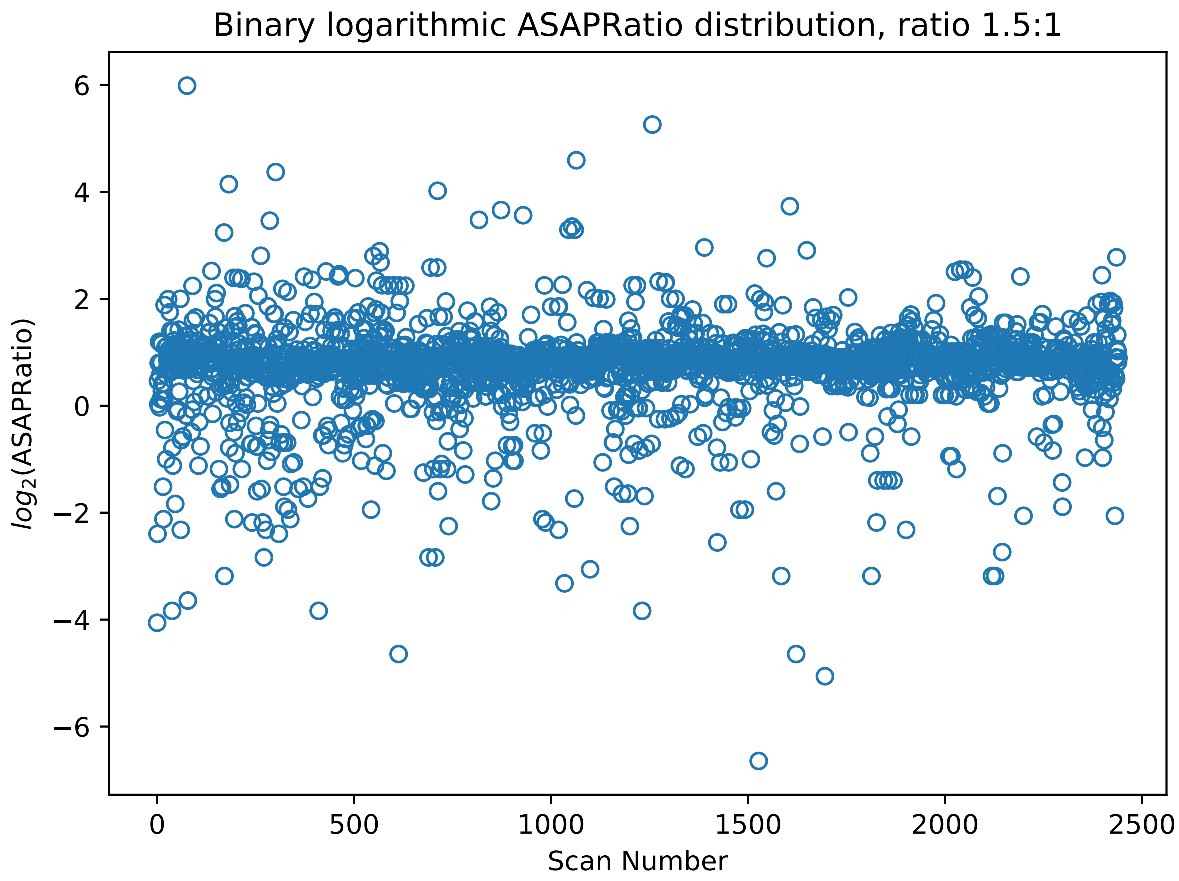
Binary logarithmic ASAPRatio distribution, ratio 1.5:1
log2(ASAPRatio)
Scan Number
6
4
2
0
−2
−4
−6
0
500
1000
1500
2000
2500

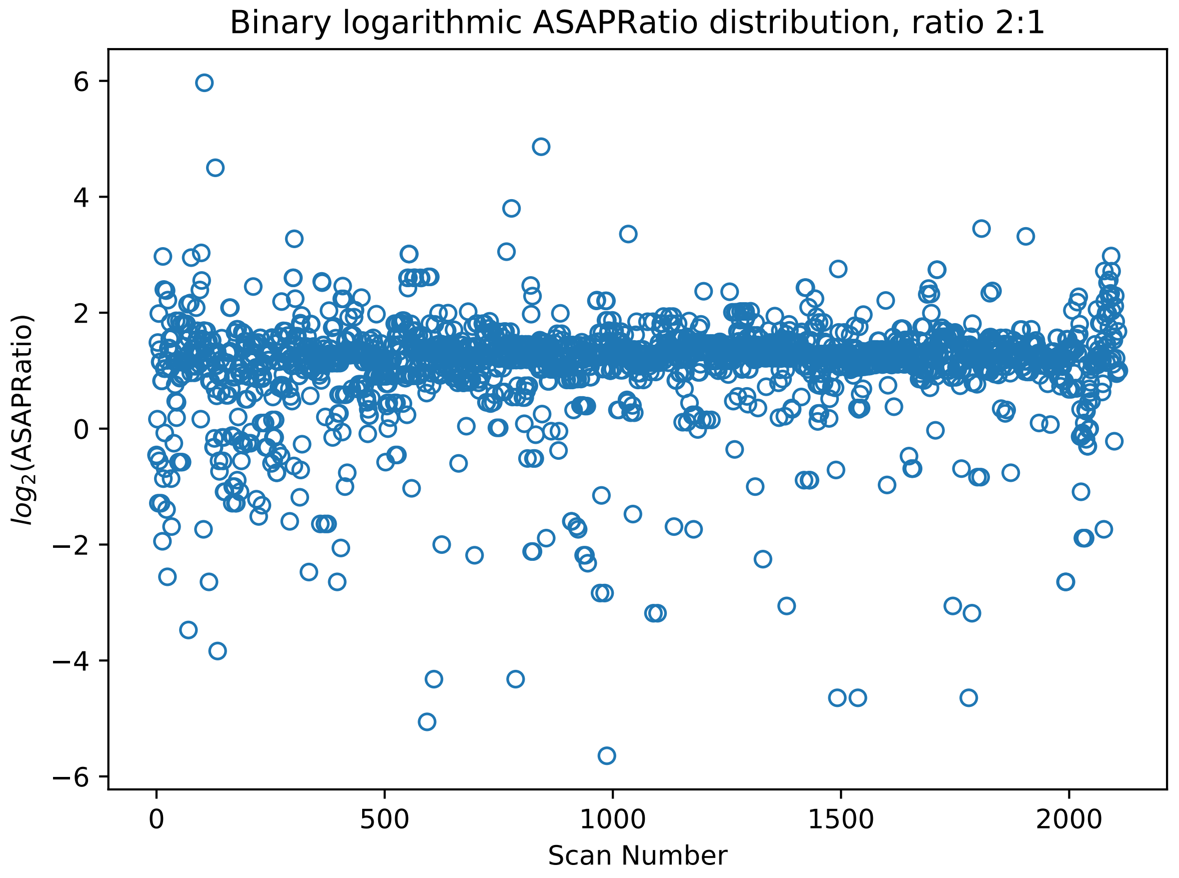
Binary logarithmic ASAPRatio distribution, ratio 2:1
log2(ASAPRatio)
Scan Number
6
4
2
0
−2
−4
−6
0
500
1000
1500
2000
