# Supplementary material for: Quality control of imbalanced mass spectra from isotopic labeling experiments
Source: BMC Bioinformatics. 2019 Nov 6;20:549. doi: 10.1186/s12859-019-3170-1 (PMC6833298; doi:10.1186/s12859-019-3170-1)
Supplement: Supplementary file 3 — Additional file 3 This is a pdf file (69KB) containing an example for controlling the quality of the spectrum with high peptide ratio. [file 12859_2019_3170_MOESM3_ESM.pdf]

Our proposed method can also assess the quality of the peptide with a higher ratio. For example, we use one 4:1 sample to test our quality control method, and get the following results,

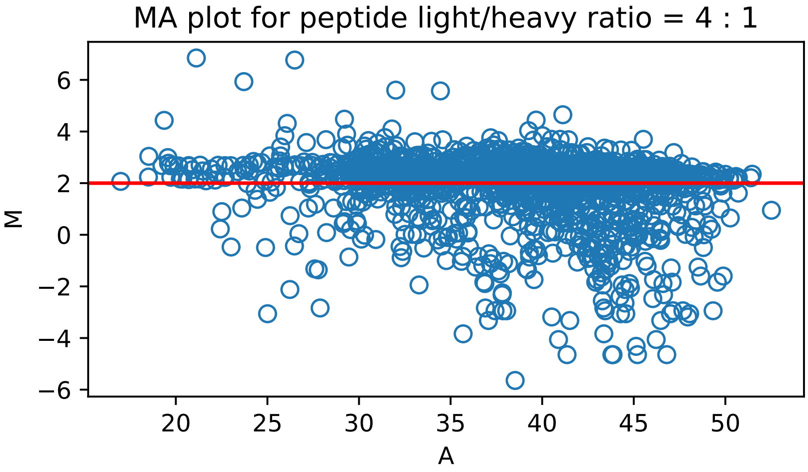

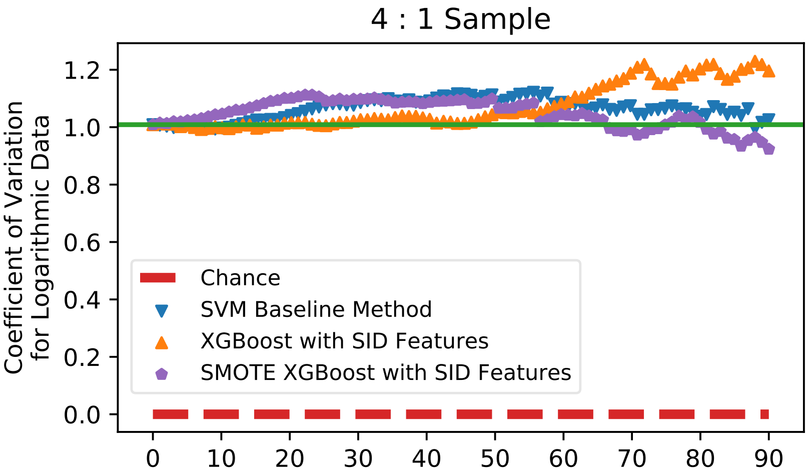

The MA plot shows that this 4:1 sample does not concentrated well, this may caused by the quantitation method. In this case study, only our proposed method makes the CV smaller. So that our method is also reliable for higher ratio samples.
